# Prolonged international normalized ratio and vascular injury at divisional level predict embolization failures of patients with iatrogenic renal vascular injuries

**DOI:** 10.1038/s41598-019-53561-z

**Published:** 2019-11-19

**Authors:** Shen-Yang Lee, Mei-Lin Wang, Yon-Cheong Wong, Cheng-Hsian Wu, Li-Jen Wang

**Affiliations:** 1 Department of Nephrology, Linkou Chang Gung Memorial Hospital, College of Medicine, Chang Gung University, Taoyuan, Taiwan; 2 Department of Medical Imaging and Intervention, Linkou Chang Gung Memorial Hospital, College of Medicine, Chang Gung University, Taoyuan, Taiwan

**Keywords:** Trauma, Radiography, Outcomes research

## Abstract

Transcatheter arterial embolization (TAE), as an alternative to surgery for iatrogenic renal vascular injury (IRVI), may have unsatisfactory outcomes. Nonetheless, there is inadequate information regarding the predictors of TAE outcomes for IRVI in the literature. The aim of this retrospective study was to investigate the predictors of TAE outcomes for IRVI. Of 47 patients, none had major complications, 17 (36.2%) patients had minor complications, and none suffered significant renal function deterioration after TAE. Technical success and clinical success were 91.5% and 93.6%, respectively. Technical failure was associated with older age, thrombocytopenia, prolonged international normalized ratio (INR) and divisional IRVI. Clinical failure was associated with kidney failure, use of steroids, prolonged INR, and divisional IRVI. In addition, prolonged INR was a significant predictor of technical failure. This implies that aggressive measures to control the INR prior to TAE are warranted to facilitate technical success, and technical success could then be validated on post-TAE images. Furthermore, divisional IRVI was a predictor of clinical failure. Thus, divisional IRVI should undergo surgery first since TAE is prone to clinical failure. The avoidance of clinical failure is validated if divisional IRVI does not need further intervention.

## Introduction

Renal vascular lesions are commonly caused by iatrogenic injuries, stab wounds, gunshots, blunt trauma, and spontaneous bleeding^1–3^. Among these, iatrogenic renal vascular injury (IRVI) is the most common (accounting for 60% of cases), and it occurs following percutaneous interventional procedures or open surgery^4^. Although IRVI could be self-limiting with no need for further treatment, severe IRVI may demand immediate surgery or transcatheter arterial embolization (TAE) to control bleeding. TAE has been used as an alternative to surgery and nephron-sparing techniques, especially in patients with multiple comorbidities who have high surgical mortality risks^5–8^.

In the literature, there are several studies on TAE for IRVI focusing on one of the following TAE outcomes, such as technical or clinical success, renal functions or complications^1,8–10^. These studies show that the majority of IRVI patients have successful outcomes, no renal function deterioration, and minor complications, if ever present^1,8–10^. In contrast, some IRVI patients undergoing TAE do have morbidities and even increased mortality^1,8–10^. Therefore, to ensure successful TAE treatment for IRVI, knowledge of potential variables contributing to poor TAE outcomes is important. Preventive measures could be employed accordingly to facilitate better results, as in many experimental studies^11–14^.

Because TAE is an invasive procedure that may lead to major or minor complications, TAE studies are usually retrospective observation studies rather than prospective or experimental studies. In contrast to variables measured from prospective or experimental studies in a standardized design or controlled setting, the variables in observation studies in a clinical retrospective study cannot be controlled but could be recorded by observation in a specific scenario. In the scenario of IRVI treated by TAE, clinical characteristics and angiographic findings are the variables we recorded to analyze the predictors of TAE results.

To our knowledge, it remains unclear which variables contribute to TAE outcomes for IRVI in the literature because of the lack of statistical analyses in the predictions of TAE outcomes stated in these observation studies^1,8–10^. Thus, this study not only assessed all TAE outcomes, including TAE success, renal function and complications but also recorded the clinical characteristics and angiographic findings of IRVI patients who underwent TAE. The aim of this study was to analyze significant predictors of TAE outcomes for IRVI based on clinical characteristics and angiographic findings using both univariate and multivariate logistic regression analyses. We hypothesize that the predictors can help select IRVI patients for TAE, carrying out appropriate preventive measures to alter the status of predictors prior to TAE, determining patients at risk for close monitoring and providing timely treatments for patients with impending TAE failures.

## Results

### Demographic and clinical features before TAE

From August 1998 to May 2013, 51 IRVI patients underwent TAE, and 4 patients were excluded due to unretrievable TAE images. The remaining 47 patients were eligible for analysis. Table 1 shows the clinical characteristics of the 47 patients with ages ranging from 26 to 78 years old. The most common pre-existing diseases were hypertension. There were 24 patients with chronic kidney disease (CKD) and 6 patients with kidney failures. Six patients had malignant tumors, including hepatoma (n = 2), bladder cancer (n = 2), renal cell carcinoma (n = 1) and lymphoma (n = 1). Interventional procedures leading to IRVI were more common than surgery. These interventional procedures included percutaneous nephrolithotomy (n = 11), percutaneous nephrostomy (n = 7), renal biopsy (n = 5), ureteroscopic stone manipulation (n = 5) and extracorporeal shock wave lithotripsy (n = 2). Anatrophic nephrolithotomy (n = 10) was the most common surgery leading to IRVI, followed by partial nephrectomy (n = 7). The most common presentation was gross hematuria. Only 4 patients had a history of shock, in contrast to 44 patients who had anemia. Eleven patients had thrombocytopenia, and 5 patients had a prolonged international normalized ratio (INR) due to warfarin in 1 patient, tacrolimus in 1 patient, and malignancy in 3 patients. Before TAE, 6 patients had the use of steroids due to renal transplantation (n = 1), skin rash after blood transfusion (n = 2), perineal erythema (n = 1), gout (n = 1) and unclear reason (n = 1).

**Table 1:** Clinical characteristics of 47 patients with iatrogenic renal vascular injury (IRVI).

Characteristics (*n* = 47)	Data
Age (years)	52 (46–63)^a^
Female: male (*n*)	20:27
Pre-existing disease	
Hypertension, *n* (%)	24 (51.1%)
Urolithiasis, *n* (%)	18 (38.3%)
Urinary tract infection, *n* (%)	8 (17.0%)
Malignancy, *n* (%)	6 (12.8%)
Renal abnormal state	
Chronic kidney disease, *n* (%)	24 (51.1%)
Kidney failure, *n* (%)	6 (12.8%)
Surgery or interventional procedures leading to IRVI	
Surgery, *n* (%)	17 (36.2%)
Interventional procedures, *n* (%)	30 (63.8%)
Clinical presentations of IRVI	
Gross hematuria, *n* (%)	31 (66.0%)
Flank pain, *n* (%)	25 (53.2%)
Shock, *n* (%)	4 (8.5%)
Hematological data	
Anemia, *n* (%)	44 (93.6%)
Thrombocytopenia, *n* (%)	11 (23.4%)
Prolonged international normalized ratio, *n* (%)	5 (10.6%)

^a^Data presented as median (interquartile range).

### Angiographic features and TAE success

Table 2 lists the angiographic findings and embolic materials of the 47 patients. IRVI occurred in the right kidney in 19 (40.4%) patients, in the left kidney in 27 (57.5%) patients, and in a transplant kidney in 1 (2.1%) patient. IRVI usually occurred at a solitary site (n = 32, 68.1%), and contrast extravasation was the most common type of IRVI (n = 33, 70.2%). Divisional IRVI was noted in 3 patients (6.4%). Coils and microcoils were the most commonly used materials (n = 41, 81.2%) for TAE. In contrast, liquid embolizers (i.e., *n*-butyl cyanoacrylate) were only used in 2 (4.3%) patients.

**Table 2:** Angiographic findings of 47 patients with iatrogenic renal vascular injury.

Angiographic findings (*n* = 47)	Data
Types of vascular injuries	
Contrast extravasation, *n* (%)	33 (70.2%)
Arteriovenous fistula, *n* (%)	16 (34.0%)
Pseudoaneurysm, *n* (%)	15 (31.9%)
Arterio-calyceal fistula, *n* (%)	4 (8.5%)
Number of vascular injury sites on angiography	
Solitary site, *n* (%)	32 (68.1%)
Multiple sites, *n* (%)	15 (31.9%)
Anatomical levels of vascular injuries	
Division, *n* (%)	3 (6.4%)
Segmental artery, *n* (%)	18 (38.3%)
Interlobar or distal artery, *n* (%)	25 (53.2%)
Capsular artery, *n* (%)	1 (2.1%)
Embolic materials	
Gelfoam, *n* (%)	9 (19.1%)
Coils or microcoils, *n* (%)	41 (87.2%)
Liquid embolizers, n (%)	2 (4.3%)

**Figure 1:**
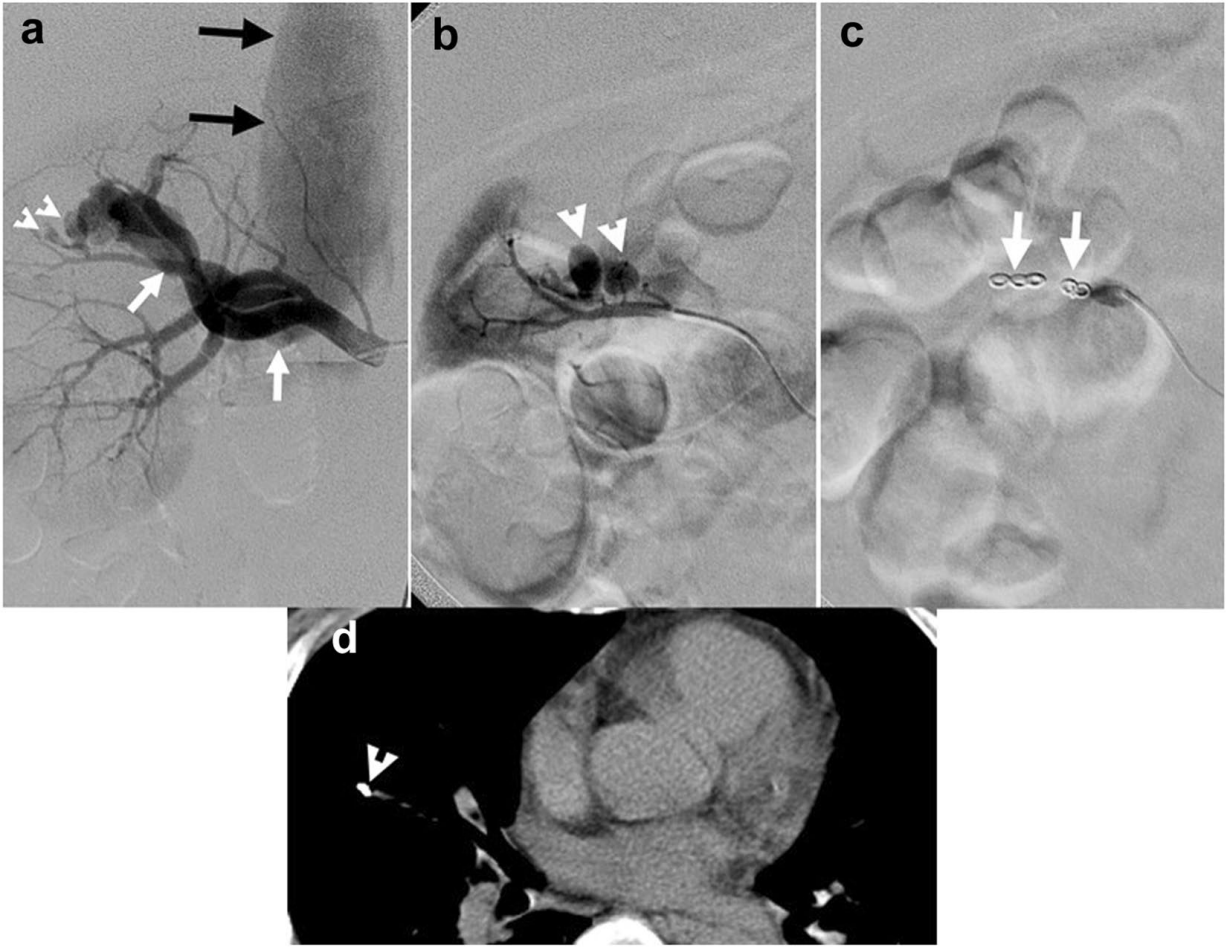
A patient presented with gross hematuria following percutaneous nephrolithotomy due to iatrogenic renal vascular injury. (**a**) Right renal angiography showing two pseudoaneurysms (arrowheads) and a large arteriovenous fistula (AVF) draining into the right renal vein (white arrows). Note the large size of the inferior vena cava (black arrows) indicating high flow of the AVF. (**b**) Right renal angiography using superselective catheterization showing pseudoaneurysms (arrowheads). (**c**) Postembolization angiography for the pseudoaneurysms showing complete cessation of the blood flow distal to the coils (arrows). (**d**) Computed tomography of the chest showing a coil (arrowhead) at the right lung after a failed attempt of embolization for the high-flow AVF.

**Figure 2:**
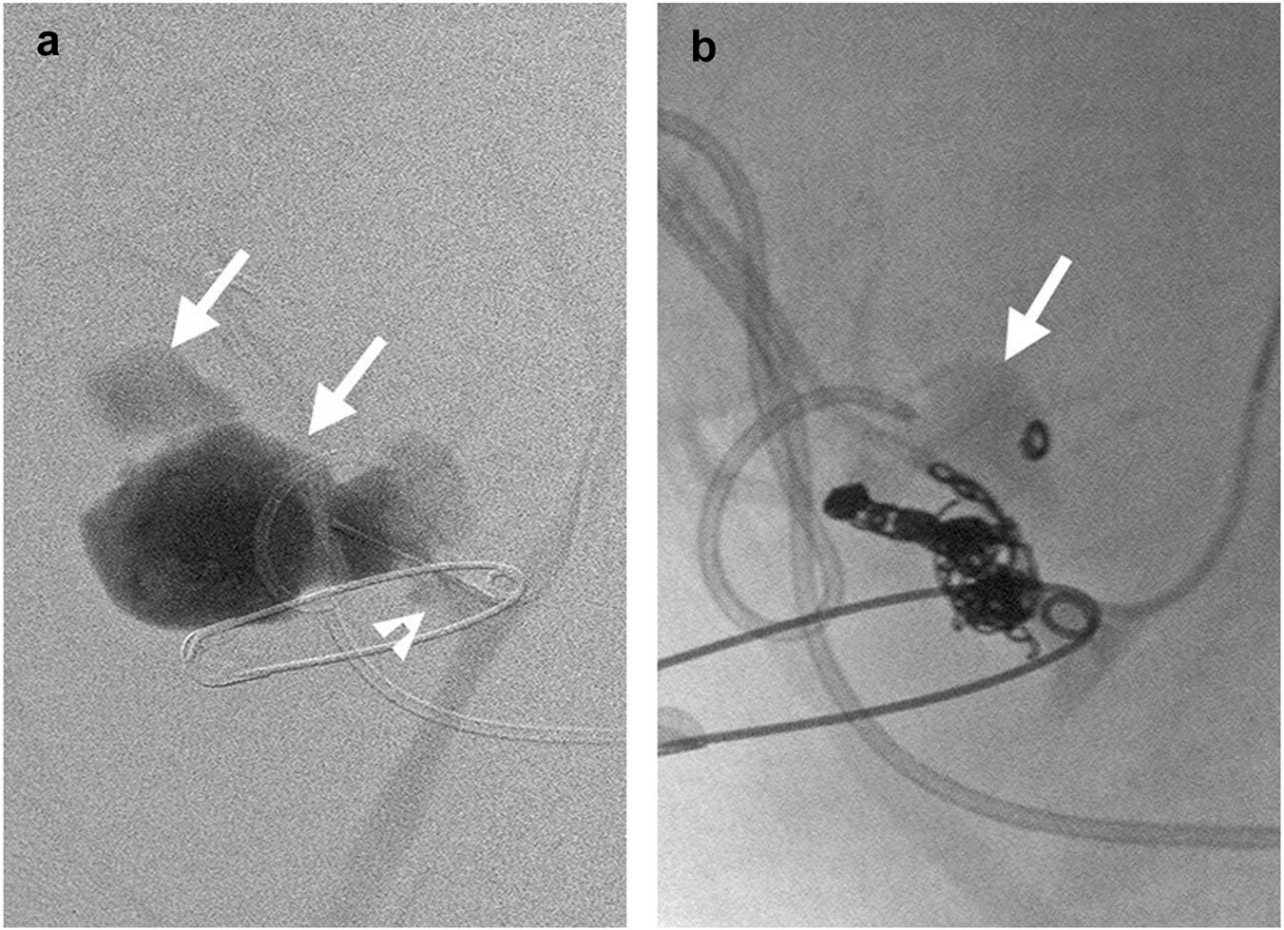
A renal transplant recipient with thrombocytopenia, a prolonged international normalized ratio and acute rejection had iatrogenic renal vascular injury following biopsy of the transplant kidney. (**a**) Renal angiography of the transplant kidney showing a large contrast extravasation (arrows) at the main artery (arrowhead). (**b**) Postembolization angiography showing persistent opacification of a smaller contrast extravasation (arrow) after deploying multiple coils.

**Figure 3:**
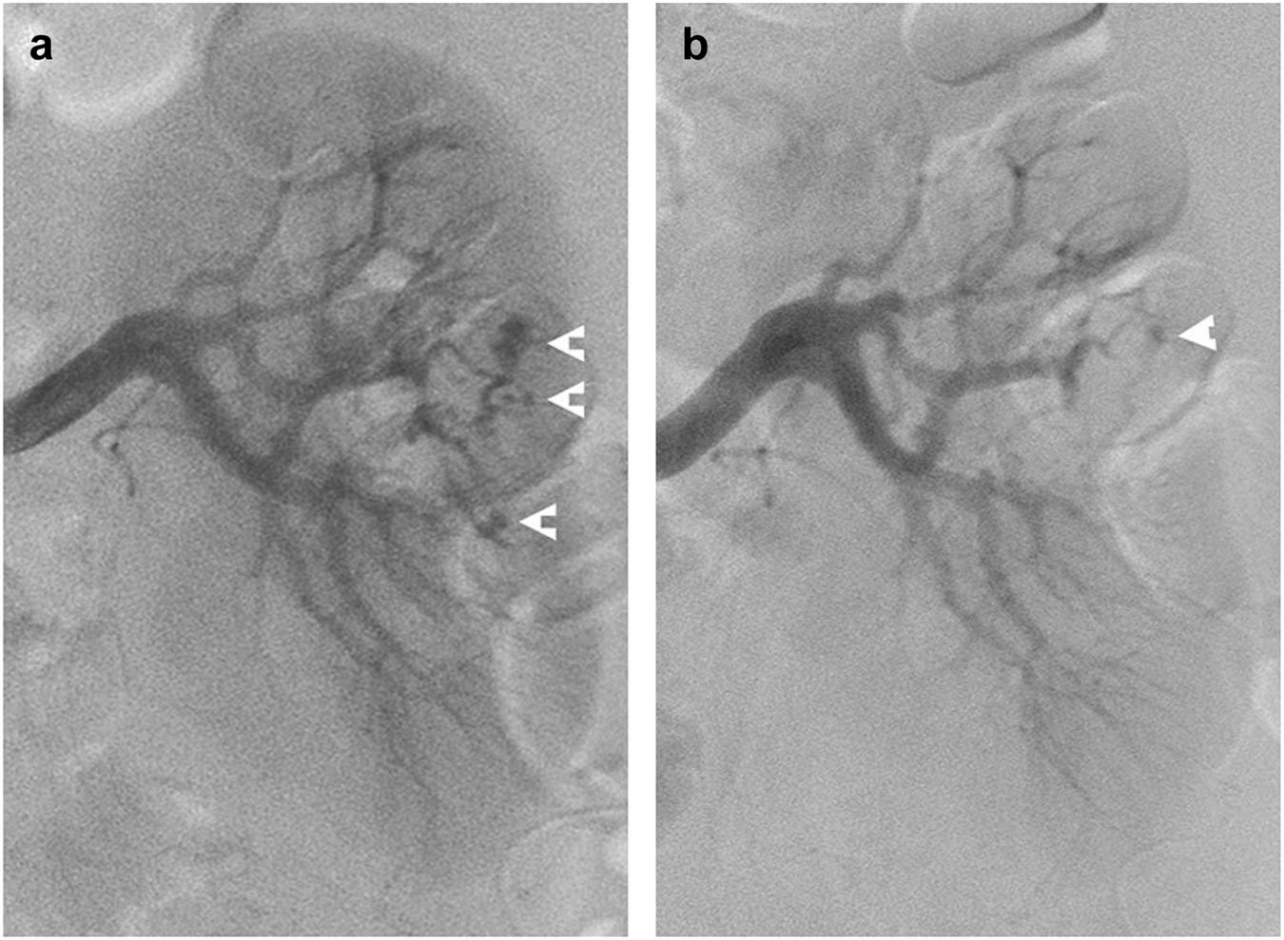
A patient with liver cirrhosis, thrombocytopenia and a prolonged international normalized ratio had renal vascular injury following partial nephrectomy, treated by transcatheter arterial embolization (TAE).(**a**) Left renal angiography showing multiple pseudoaneurysms (arrowheads). (**b**) Post-TAE image showing a small residual pseudoaneurysm (arrowhead). Gross hematuria resolved following TAE, and the patient had an uneventful course, requiring no further intervention or surgery.

Overall, the technical success rate was 91.5% (43 of 47 patients), and the clinical success rate was 93.6% (44 of 47 patients). Only five patients experienced TAE failures, and their clinical characteristics and angiographic findings are summarized in Table 3. Patients #1 and #2 had both technical and clinical failures. Patient 1 had a high-flow AVF and pseudoaneurysms (Fig. 1). After successful embolization of the pseudoaneurysms, the first deployed coil for embolization of the high-flow AVF immediately migrated to the right pulmonary arterial branch. This patient underwent a subsequent nephrectomy to control bleeding. Patient #2 was a renal transplant recipient who had an IRVI in the transplant kidney due to a biopsy (Fig. 2). The post-TAE angiographic images showed partial obliteration of the contrast extravasation at the main renal artery of the transplant kidney, indicative of technical failure. Inotropic support and blood transfusion were undertaken due to unstable vital signs. Persistent oozing and infarct of the transplant kidney was found, and the patient expired during an attempted nephrectomy. Patients #3 and #4, with prolonged INR and thrombocytopenia, had clinical success despite technical failures evidenced by small residual pseudoaneurysms shown on post-TAE images (Fig. 3). Patient #5 had double cancers, including prostate cancer with bony metastases and recurrent bladder cancer with perivesical invasion. He had clinical failure despite technical success of TAE, and he underwent percutaneous nephrostomy for infected obstructive uropathy following TAE and ultimately expired due to severe urosepsis.

**Table 3:** Clinical characteristics and angiographic findings of the 5 patients with transcatheter arterial embolization (TAE) failure.

Patient	Sex/age range (median)	Clinical and angiographic findings				TAE failures			
		Thrombocytopenia	Prolonged international normalized ratio	Types of vascular injuries	Division level	Technical /clinical failure	Post-TAE images	Further surgery or intervention	Survival
1	2 females and 3 males/ 31–73 years old (median: 45 years old)	No	No	High-flow arteriovenous fistula and pseudoaneurysms	Yes	Both failures	Untargeted embolization of a branch of the pulmonary artery	Nephrectomy	Yes
2		Yes	Yes	Large contrast extravasation	Yes	Both failures	Faint opacification of residual small contrast extravasation	Nephrectomy	No
3		Yes	Yes	Multiple contrast extravasations	No	Technical failure	Residual small contrast extravasations	None	Yes
4		Yes	Yes	Multiple pseudoaneurysms	No	Technical failure	A small residual pseudoaneurysm	None	Yes
5		Yes	Yes	Multiple contrast extravasations	No	Clinical failure	No more contrast extravasations	None	No

### Associated variables and predictors of TAE failures

Table 4 shows the relationships between clinical/angiographic characteristics and TAE success. Age, thrombocytopenia, prolonged INR and divisional IRVI showed statistically significant differences between technical success and failure patients. Prolonged INR, divisional IRVI, kidney failure and use of steroids were associated with clinical failure (all *p* < 0.05). In contrast, other variables were not associated with technical or clinical failures. Further multivariate logistic regression analysis (Table 5) showed that prolonged INR was a significant predictor of TAE technical failure and divisional IRVI, of TAE clinical failure.

**Table 4:** Clinical and angiographic characteristics of patients with iatrogenic renal vascular injury (IRVI) associated with technical and clinical success of transcatheter arterial embolization.

Variables	Technical success			Clinical success		
	Yes (*n* = 43)	No (*n* = 4)	p value	Yes (*n* = 44)	No (*n* = 3)	p value
Age (years old)	54.65 ± 13.41	41.25 ± 8.66	0.047*	53.77 ± 13.18	49.67 ± 21.39	0.557
Female, *n* (%)	18 (41.9 %)	2 (50.0 %)	1.000	19 (43.2 %)	1 (33.3 %)	1.000
Pre-existing diseases						
Hypertension, *n* (%)	23 (48.9 %)	1 (25.0 %)	0.348	22 (50.0 %)	2 (66.7 %)	1.000
Urolithiasis, *n* (%)	17 (39.5 %)	1 (25.0 %)	1.000	17 (38.6 %)	1 (33.3 %)	1.000
Urinary tract infection, *n* (%)	8 (18.6 %)	0 (0.0 %)	1.000	8 (18.2 %)	0 (0.0 %)	1.000
Malignancy, *n* (%)	4 (9.3 %)	2 (50.0 %)	0.074	5 (11.4 %)	1 (33.3 %)	0.343
Surgery leading to IRVI, *n* (%)	16 (37.2 %)	1 (25.0 %)	1.000	17 (38.6 %)	0 (0.0 %)	0.292
Clinical presentations						
Gross hematuria, *n* (%)	29 (67.4%)	2 (50.0%)	0.597	29 (65.9%)	2 (66.7%)	1.000
Flank pain, *n* (%)	23 (53.5%)	2 (50.0%)	1.000	24 (54.6%)	1 (33.3%)	0.593
Shock, *n* (%)	4 (9.3%)	0 (0.0%)	1.000	4 (9.1%)	0 (0.0%)	1.000
Hematological data						
Anemia, *n* (%)	40 (95.3%)	4 (100.0%)	1.000	41 (93.2%)	3 (100.0%)	1.000
Thrombocytopenia, *n* (%)	8 (18.6 %)	3 (75.0 %)	0.035*	9 (20.5 %)	2 (66.7 %)	0.132
Prolonged international normalized ratio, *n* (%)	2 (4.7%)	3 (75.0%)	0.002*	3 (6.8%)	2 (66.7%)	0.027^*^
Renal abnormal state						
Chronic kidney disease	22 (51.2 %)	2 (50.0 %)	1.000	22 (50.0 %)	2 (66.7 %)	1.000
Kidney failure	5 (11.6 %)	1 (25.0 %)	0.432	4 (9.1 %)	2 (66.7 %)	0.039*
Use of steroids before TAE	5 (11.6 %)	1 (25.0 %)	0.432	4 (9.1 %)	2 (66.7 %)	0.039*
Experience levels of radiologists						
Years of experience	12.98 ± 5.44	10.00 ± 6.28	0.225	12.89 ± 5.41	10.33 ± 7.57	0.344
≧10 years of experience	31 (72.1 %)	1 (25.0 %)	0.089	31 (70.5 %)	1 (33.3 %)	0.235
Angiographic characteristics						
Contrast extravasation, *n* (%)	30 (69.8%)	3 (75.0%)	1.000	30 (68.2%)	3 (100%)	0.544
Arteriovenous fistula, *n* (%)	15 (34.9%)	1 (25.0%)	1.000	15 (34.1%)	1 (33.3%)	1.000
Pseudoaneurysm, *n* (%)	14 (32.6%)	1 (25.0%)	1.000	15 (34.1%)	0 (0.0%)	0.541
Arterio-calyceal fistula, *n* (%)	4 (9.3%)	0 (0.0%)	1.000	4 (9.1%)	0 (0.0%)	1.000
Multiple IRVI sites, *n* (%)	12 (27.9%)	3 (75.0%)	0.089	13 (29.6%)	2 (66.7%)	0.235
Division, *n* (%)	1 (2.3%)	2 (50.0%)	0.016^*^	1 (2.3%)	2 (66.7%)	0.008^*^
Segmental artery, *n* (%)	18 (41.8 %)	0 (0.0 %)	0.283	18 (40.9 %)	0 (0.0 %)	0.276
Interlobar or distal artery, *n* (%)	23 (53.5 %)	2 (50.0 %)	1.000	24 (54.5 %)	1 (33.3 %)	0.593
Capsular artery, *n* (%)	1 (2.3 %)	0 (0.0 %)	1.000	1 (2.3 %)	0 (0.0 %)	1.000
Embolic materials						
Gelfoam	8 (18.6 %)	1 (25.0 %)	1.000	8 (18.2 %)	1 (33.3 %)	0.480
Coils/microcoils	38 (88.4 %)	3 (75.0 %)	0.432	39 (88.6 %)	2 (66.7 %)	0.343
Liquid embolizers	2 (4.7 %)	0 (0.0 %)	1.000	2 (4.5 %)	0 (0.0 %)	1.000

^*^Statistically significant difference.

**Table 5:** Results of multivariate logistic regression analysis of significant predictors of TAE technical and clinical failures.

TAE outcome	Variables	Odds ratio (95 confidence interval)	p value
Technical failure	Prolonged international normalized ratio	61.5 (4.3–889.3)	0.003*
Clinical failure	Vascular injury at division level	86.0 (3.8–1934.8)	0.003*

*Statistically significant difference.

### TAE complication and renal function change analysis

None of the 47 patients had major complications. Seventeen (36.2%) patients had minor complications, including untargeted embolization in 1 (2.1%) patient and postembolization syndrome in 16 (34.0%) patients. Untargeted embolization to a small branch of the right pulmonary artery caused no symptoms, and the patient required no therapy. All patients with postembolization syndrome subsided after nominal medicine therapy. No clinical characteristics and angiographic findings were associated with minor complications (all p > 0.05).

Before TAE, all 47 patients had renal function data, and their median estimated glomerular filtration rate (eGFR) was 58.56 ml/min/1 with an interquartile range (IQR) of 37.49–74.20 ml/min/1.73 m^2^ from the 25th to 75th percentile. Thirty-seven (78.7%) patients had renal function data at a median of 173 days after TAE, and their median eGFR was 59.00 ml/min/1.73 m^2^ with an IQR of 42.66–72.21 ml/min/1.73 m^2^. There was no statistically significant difference between the eGFR before and after TAE (p > 0.05).

## Discussion

IRVI is a potentially life-threatening condition occurring after kidney surgery or interventional procedures that requires an immediate survey and management of renal hemorrhage. TAE is an effective measure to control bleeding for IRVI, with an expectedly high success rate for the majority of patients. Recently, several studies of TAE for IRVI have reported technical and clinical success rates of 87–100% and 89–98%, respectively^1,2,8,15^. Our study showed high technical and clinical success rates of 91.5% and 93.6%, respectively, comparable to previous studies. In contrast, TAE failures did occur in a small percentage of patients who required further surgery or intervention.

The present study showed that prolonged INR was a significant predictor of technical failure in multivariate analyses after controlling for other variables. A variety of conditions (liver cirrhosis, renal transplant rejection, cancer, or warfarin use) are associated with prolonged INR, and severe coagulopathy is also a contraindication to surgery^16,17^. The difficulty of achieving technical success in IRVI patients could be explained by the hemostasis and coagulation mechanism of TAE. However, the technical failures of TAE may not always result in clinical failures, and 2 coagulopathic patients having technical failures by small residual lesions on the post-TAE images in this study ultimately had clinical success. A case series of 20 patients with 23 spontaneous bleeding sites due to anticoagulant therapy also reported clinical success of TAE at 85%^18^. Thus, for IRVI patients with coagulopathies, TAE might still be acceptable as an alternative to surgery, although aggressive control of coagulopathy prior to TAE should be implemented whenever possible^19–21^.

This study showed that divisional IRVI was a significant predictor of clinical failure of TAE. A retrospective study of renal vascular injury also revealed that divisional IRVI had a worse outcome than segmental artery injury^22^. There are several explanations for this finding. First, the pressure and flow rate are much higher at the divisional level than at distal sites^23,24^. It is thus much more difficult to achieve hemostasis in divisional IRVI than at other levels. A few studies using interlocking detachable coils have been recently reported with successful TAE results^25,26^. Unfortunately, these specifically designed coils have not yet been available at the time of TAE for divisional IRVI patients with high-flow AVF in this study. Second, embolization at the divisional level usually inevitably results in renal ischemia or infarction in a larger portion of the whole kidney. Furthermore, ischemia or infarct may cause complications, such as abscess formation and sepsis, and thus increase the risk of clinical failure. Thus, close monitoring of clinical conditions following TAE is necessary for divisional IRVI patients to provide timely and appropriate intervention or surgery whenever possible.

There are few complications of renal TAE, and most are minor. The reported major complication rate of renal TAE ranged from 0.6–12% with a suggested threshold of 6%^7^. However, the occurrence of complications is not associated with the type of embolic material, as noted in a previous report^1^. This study also showed that there is no association between clinical and angiographic characteristics and TAE complications. Sam *et al*. reported on three patients with complications due to untargeted embolization without clinical symptoms and renal functional loss, and they also classified these complications as minor^8^. Our study shows that postembolization syndrome is the most common complication of renal TAE. Similarly, Song *et al*. reported a postembolization syndrome rate of 22% in 36 patients undergoing TAE for IRVI^1^. It is generally accepted that postembolization syndrome reflects reactions to renal ischemic or infarct changes following blood flow cessation. Nonetheless, postembolization syndrome is a self-limited condition that can be treated with medicine^1^.

Renal TAE usually has no permanent renal function deterioration effect, as shown in this study and in the literature^1,4,8,27^. Song *et al*. reported an increase in serum creatinine levels in 14 of 17 patients (82.4%) with pre-TAE renal insufficiency due to contrast-induced nephropathy, which is transient and reversible^1^. With the development of superselective catheterization techniques used in TAE, cessation of the blood flow could be achieved with minimal renal parenchyma devascularization by deploying embolic materials proximal to the bleeding sites^1^. Therefore, this study using superselective TAE showed that there was no significant difference between the renal functions before and after TAE.

The present study has several limitations. First, this was a retrospective study with a limited number of patients. Although 2 patients using liquid embolizers for TAE had both technical and clinical success, the statistical analysis result showed no association of liquid embolizers and TAE outcomes because of low statistical power by the small case number. Second, TAE for IRVI was performed by 16 certified interventional radiologists who constituted an urgent interventional service team and were in charge of urgent embolization procedures following interventional principles and standard procedures of TAE. Although there could be interoperator variance for TAE outcomes among different radiologists in theory, the high success rates of TAE in this study ensures the quality and efficacy of TAE by this team regardless of individual variance. Third, we did not use machine learning methods for predicting TAE outcomes, which might be useful to analyze the relationships between TAE outcomes and clinical/angiographic characteristics if there was a sufficiently large number of IRVI patients^28,29^. Nonetheless, there were 2–3 IRVI patients on average per year, and a prospective study design would also be futile for enrolling a sufficient number of patients for this study. Finally, the data have been collected since 1998, over a period of 15 years, and the technology of TAE has developed and evolved over time, and this could possibly affect the final outcome in some cases.

In conclusion, this study shows that TAE has a high success rate, a low major complication rate and no significant renal function deterioration for IRVI patients. However, prolonged INR is a significant predictor of TAE technical failure, and an effort to correct coagulopathy before TAE should be made whenever possible. This study also shows that divisional IRVI is a significant predictor of TAE clinical failure. Thus, divisional IRVI patients should undergo surgery first since TAE is prone to clinical failure and may ultimately lead to mortality and morbidity. If surgery was contraindicated for a divisional IRVI patient, the possibility of TAE clinical failure should be fully explained to the patient, the patient’s family and to referring physicians. Close monitoring of vital signs should be performed both during and after TAE for divisional IRVI patients; if TAE clinical failure did occur, another intervention or surgery (e.g., nephrectomy) should be provided as soon as possible.

## Methods

### Subjects

This retrospective study was approved by our institutional review board, and informed consent was waived for retrospective studies according to the policy of our institutional review board. This study analyzed all IRVI patients undergoing TAE at our hospital between August 1998 and May 2013. For each patient, age, sex, pre-existing diseases, presence of CKD and renal failure, surgical or interventional procedures leading to IRVI, clinical presentations (gross hematuria, flank pain, and shock), and use of steroids before TAE were recorded via medical chart review. CKD was defined as abnormal kidney structure or function for more than 3 months, with implications for health^30^. For patients with eGFR <15 ml/min/1.73 m^2^, kidney failure was considered^30^. Shock was considered when the shock index (i.e., heart rate/systolic blood pressure) was ≥0.9^31^. Hematological data, including hemoglobin level, platelet count and INR, were also recorded. Anemia was defined by the World Health Organization criteria: hemoglobin less than 13 g/dl in males and 12 g/dl in females^32^. Thrombocytopenia was defined as a platelet count below 15 × 10^9^/L^33^. An INR > 1.4 was considered as prolonged INR^34^.

### Renal angiography and embolization procedure

Before undergoing renal angiography and TAE, informed consent was obtained from each patient after a detailed explanation of the procedures, possible complications and contrast medium allergy of renal angiography and TAE. Renal angiography and the TAE of each IRVI patient were performed in accordance with the institutional regulations by 16 certified radiologists who had 5–25 years of experience in interventional procedures. The standard procedures of TAE for IRVI patients included several steps. First, renal angiography was performed to assess IRVI locations and types and to demonstrate the roadmaps of bleeding arteries for subsequent TAE. Second, TAE with superselective catheterization of the bleeding vessels was performed with the deployment of appropriate embolizers to stop the blood flow of the bleeding arteries. Third, post-TAE angiography was performed immediately after completion of TAE, which served as a validation procedure by confirming cessation of blood flow of the bleeding arteries.

**Figure 4:**
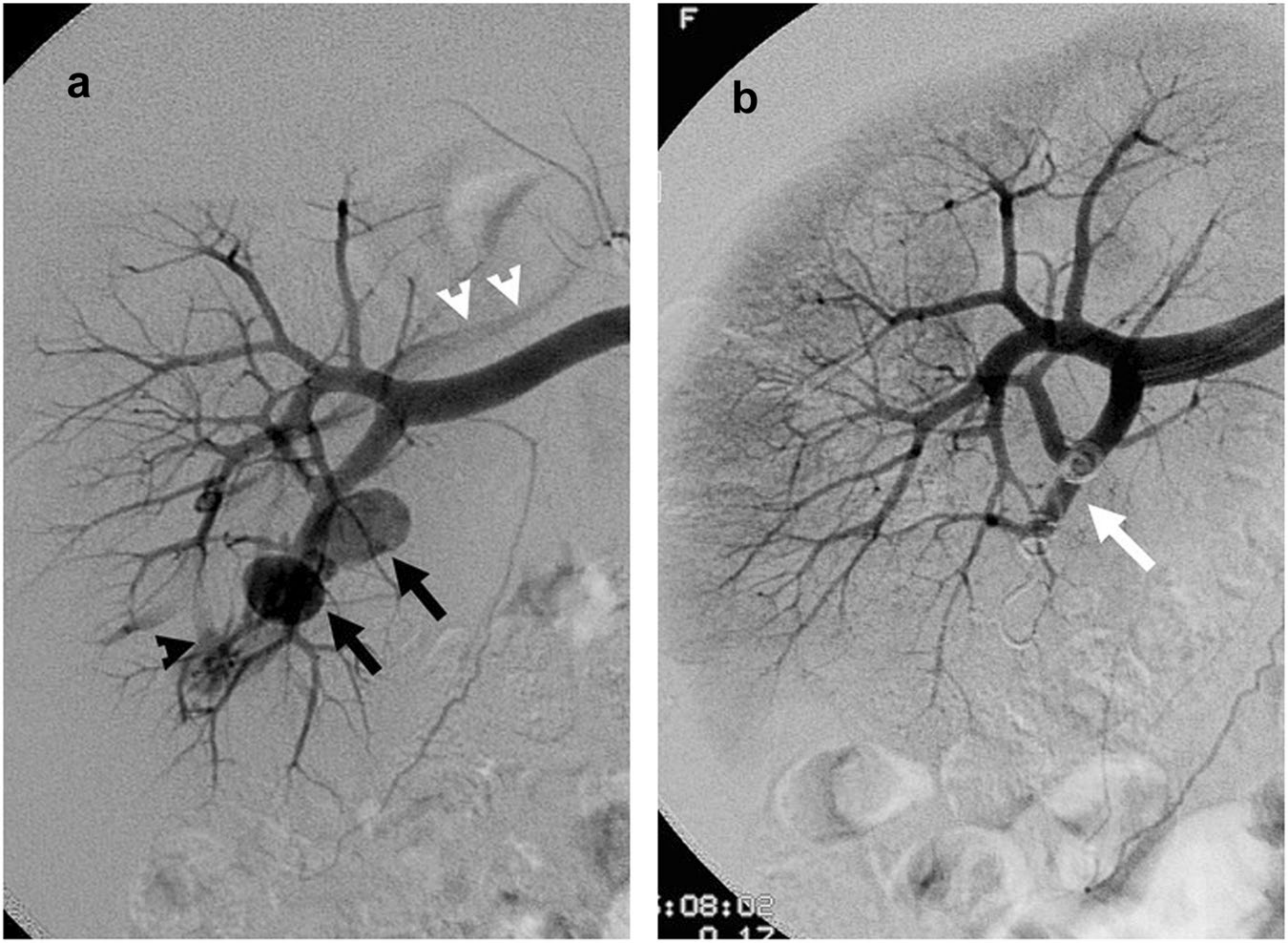
A patient with right renal vascular injury after percutaneous nephrostomy was successfully treated by transcatheter arterial embolization (TAE). (**a)** Right renal angiography showing contrast extravasations (black arrows) at the segmental artery of the right renal lower pole. Note the presence of an arteriovenous fistula (black arrowhead) draining into the right main renal vein (white arrowhead). (**b)** Renal angiography after TAE showing nonvisualization of contrast medium extravasation after embolization by coils (white arrow).

On renal angiography, imaging characteristics of IRVI of each patient were recorded by type, site number and level. The types of IRVI were categorized by contrast extravasation, pseudoaneurysm, arteriovenous fistula (AVF), and arterio-calyceal fistula (Fig. 4). The site number was categorized as solitary or multiple sites. The levels of IRVI were recorded as divisional (i.e., at the main artery or divisions), segmental, interlobar or distal, and capsular artery levels. For IRVI patients showing contrast extravasation and pseudoaneurysm on pre-TAE renal angiography, TAE was performed with the aim of stopping the blood flow to the sites of contrast extravasation and pseudoaneurysm, and post-TAE renal angiography was used to confirm the disappearance of contrast extravasation and pseudoaneurysm after TAE. For IRVI patients showing AVF and arterio-calyceal fistula on pre-TAE angiography, TAE was performed with the aim of occluding the arterial side accounting for the AVF and arteriovenous fistula, followed by post-TAE angiography to confirm the disappearance of AVF and arterio-calyceal fistula. During TAE, superselective catheterization of the bleeding vessels as distal as possible was typically performed with the use of a 3-F microcatheter, followed by deployment of embolic materials immediately proximal to the bleeding sites to limit the embolization effect to the bleeding sites and to minimize adjacent renal parenchymal devascularization^8^. The embolic materials were recorded as gelfoam, coils/microcoils, and liquid embolizers. The use of gelfoam, coils and microcoils for TAE was covered according to the reimbursement policy of government insurance. If the embolic materials were not covered by insurance policy, then the patients had to decide whether or not they were willing to pay for the bills. The choice of embolic materials was thus dependent on the discretion of operators, availability of embolic materials at the time of TAE and the patients’ decision.

### TAE success evaluation

Post-TAE images and the clinical course of all patients following TAE were reviewed to validate their technical and clinical success. Technical success was defined as complete occlusion of the bleeding vessels on post-TAE angiography^1,8^. Clinical success was defined as not needing further surgery or intervention after TAE^7,8^.

### TAE complication and renal function assessment

TAE complications were recorded and categorized into major and minor complications after review of the clinical course of each patient after TAE^7^. Major complications were considered for TAE-related death, permanent adverse sequelae, or complications requiring therapy or hospitalization^7^. For each patient, renal functions were recorded as eGFR using the modification of diet in renal disease formula before and after TAE via medical chart review^35^. The pre-TAE renal function was selected as the last data within 1 month before TAE. The post-TAE renal function of each patient was recorded as the last data within 1 year after TAE to avoid confounders such as contrast-induced nephropathy and shock^8^.

### Statistics

All variables were summarized using descriptive statistics, which were expressed as median and IQR (i.e., 25th to 75th percentile) or mean and standard deviation for continuous variables as well as numbers and proportions for categorical variables. The associations of continuous variables with TAE outcomes were analyzed using the Mann-Whitney U tests and of categorical variables using the Chi-square or Fisher’s exact tests. Multivariate logistic regression analyses with stepwise selections were further analyzed for predictors of TAE failures. The renal functions before and after TAE were compared using the Wilcoxon signed rank test. All statistical tests were two-tailed with significance levels at α-values of 0.05 using Medcalc (version 16.4.3, Ostend, Belgium).

## Data Availability

The datasets analyzed during the current study are available from the corresponding author on reasonable request.
